# Male dominance status regulates odor-evoked processing in the forebrain of a cichlid fish

**DOI:** 10.1038/s41598-019-41521-6

**Published:** 2019-03-25

**Authors:** Alexandre A. Nikonov, Karen P. Maruska

**Affiliations:** Department of Biological Sciences, Louisiana State University, 202 Life Sciences Bldg., Baton Rouge, LA 70803 USA

## Abstract

The ability to identify odors in the environment is crucial for survival and reproduction. However, whether olfactory processing in higher-order brain centers is influenced by an animal’s physiological condition is unknown. We used *in vivo* neuron and local field potential (LFP) recordings from the ventral telencephalon of dominant and subordinate male cichlids to test the hypothesis that response properties of olfactory neurons differ with social status. Dominant males had a high percentage of neurons that responded to several odor types, suggesting broad tuning or differential sensitivity when males are reproductively active and defending a territory. A greater percentage of neurons in dominant males also responded to sex- and food-related odors, while a greater percentage of neurons in subordinate males responded to complex odors collected from behaving dominant males, possibly as a mechanism to mediate social suppression and allow subordinates to identify opportunities to rise in rank. Odor-evoked LFP spectral densities, indicative of synaptic inputs, were also 2–3-fold greater in dominant males, demonstrating status-dependent differences in processing possibly linking olfactory and other neural inputs to goal-directed behaviors. For the first time we reveal social and reproductive-state plasticity in olfactory processing neurons in the vertebrate forebrain that are associated with status-specific lifestyles.

## Introduction

An animal’s ability to detect and correctly identify social signals is crucial for making appropriate context-dependent behavioral decisions. In vertebrates, odorants are detected by olfactory receptor neurons (ORNs), the axons of which form the olfactory nerve and project to mitral cells within specific glomerular fields of the olfactory bulb^1–4^. Information from the olfactory bulb is then sent to target forebrain processing centers, where it is integrated with other information to mediate behaviors^5–7^. While recent work has revealed insights on odor coding at levels above the olfactory bulb in mammals^8,9^, less is known about how this ascending olfactory information is processed in fishes, the largest and most diverse group of vertebrates.

Chemoreception (olfaction, taste, and common chemical sense) is a phylogenetically old group of senses and perhaps most salient in fishes, which live in an aquatic mixture of soluble odorants used to detect food, evade predators, locate habitats, and identify conspecifics for social interactions including territoriality, mating, and parental care^10,11^. Chemosensory communication during aggression and reproduction is an important component of fish sociality. For example, information on male dominance status in cichlids and female reproductive condition in goldfish are conveyed via released chemical molecules and odor mixtures that are received by the olfactory system^12–14^. Despite the widespread importance of this olfactory information to survival and reproduction, our knowledge of how neurons in decision centers process biologically-relevant odors is extremely limited.

The reception of sensory information in specific behavioral contexts can also be modulated by an animal’s internal physiological, hormonal, and motivational states. As a result, an individual may respond very differently to the same social signals received at distinctly different times. Evidence for hormonal or reproductive-state modulation of sensory processing is documented for audition^15,16^, vision^17–19^, and olfaction^20–23^. In the olfactory system, support for modulation exists primarily at the level of the olfactory epithelium and olfactory bulb, but whether there are also differences in response properties of neurons within higher processing centers is relatively unexplored in any taxa. Given that chemosensory communication was preserved over evolutionary time and is widespread across the animal kingdom, particularly in reproductive contexts, examining how olfactory abilities change with social and reproductive state deserves attention. This is particularly relevant for seasonally breeding species as well as those living in dominance hierarchies, where status position is critically linked to health, survival, and reproductive fitness^24,25^. Are shifts in priorities associated with an animal’s current lifestyle associated with plasticity in detecting and processing context-dependent olfactory information?

To test whether an animal’s social rank and reproductive state influences neuronal response properties to socially-relevant odors, a species that shows natural social plasticity and relies on olfaction for behavioral decisions is needed. The African cichlid fish *Astatotilapia burtoni* is ideally suited because males exist in dominance hierarchies in which dominant individuals aggressively defend territories and reproduce with females, while subordinates do not hold territories, shoal with females, and have limited mating opportunities. Social rank also determines male reproductive physiology, with dominant individuals having an up-regulated reproductive axis from the brain to the testis compared to subordinate males [reviewed in^26–29^]. Males also use many sensory modalities during social interactions^30–32^ and olfaction in particular is involved in both inter- and intra-sexual communication^33^. For example, dominant males increase their urination in the presence of both receptive females and rival males, and gravid sexually-receptive females increase urination towards courting dominant males^33,34^. Further, female-released chemical signals provide non-redundant information to males via olfaction (not taste), increase male courtship behaviors, and are associated with differential neural activation patterns in the brain^35^. In male *A*. *burtoni*, therefore, olfaction plays a key role in controlling both social rank and reproductive opportunity, raising the possibility of status-specific olfactory processing abilities.

The goal of this study was to test the hypothesis that odor-evoked response properties of the ventral telencephalon differs with male social rank. We used *in vivo* single-/multi-unit (one or multiple neurons) and local field potential (LFP) recordings to compare odor-evoked responses in dominant and subordinate males to both complex odors and pure odorants. To our knowledge, we demonstrate social status-specific differences in forebrain olfactory processing for the first time in any vertebrate. This plasticity associated with social position and reproductive state in a fish also leads to the testable hypothesis that it may be a conserved olfactory processing mechanism across vertebrates.

## Results

### *In Vivo* Odor-Evoked Response Properties of Neurons in the Cichlid Ventral Telencephalon

**Figure 1:**
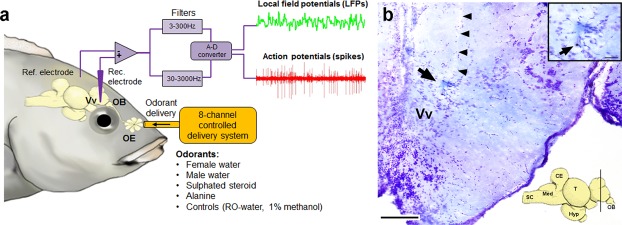
*In vivo* odor-evoked single-/multi-units and local field potentials were recorded from the ventral telencephalon of the cichlid *A*. *burtoni*. (**a**) Differential recordings were made from the telencephalon while test odors were delivered to the ipsilateral olfactory epithelium (OE) via an 8-channel controlled gravity perfusion system. Action potentials and local field potentials (LFPs) were recorded simultaneously from the same recording electrode onto separate channels through the use of different band-pass filters. EOG recordings not shown. (**b**) Nissl-stained cross section through the telencephalon showing a representative electrolytic lesion (arrow) of the recording site in the dorsal portion of the ventral nucleus of the ventral telencephalon (Vv) (scale bar = 100 µm). The electrode track (arrowheads) can also be seen. Inset at top right shows a higher magnification of the lesion where a gold ball (~5 µm dia.) from the electrode tip was deposited from the delivered electric current and is visible (arrow) (scale bar = 25 µm). Inset at bottom shows a sagittal brain with approximate location of the cross section indicated. CE, cerebellum; Hyp, hypothalamus; Med, medulla; OB, olfactory bulb; SC, spinal cord; T, tectum.

To test for social status differences in odor-evoked responses of a forebrain center in the cichlid, we recorded *in vivo* single-/multi-unit responses and local field potentials (LFPs) from the ventral telencephalon in dominant and subordinate male *A*. *burtoni*. The target recording location was the ventral nucleus of the ventral telencephalon (Vv), regions of which are homologous in part to the lateral septum and striatal external globus pallidus of mammals^36–38^. The Vv was chosen because it is an important olfactory processing region in fishes^5,7,39^, as well as a decision center shared by the social behavior network and mesolimbic reward pathways that is important for many social behaviors^40^. We recorded the full suite of responses from a total of 23 neurons (11 neurons in 7 subordinate males; 12 neurons in 7 dominant males; 1–2 neurons per fish), and examined odor-evoked responses to controlled delivery of two control (RO-water, 1% methanol) and four test odors (male water, female water, sulphated sex-steroid, and alanine) applied to the ipsilateral olfactory epithelium (Fig. 1a) (see methods). Male water and female water were complex odor mixtures collected from behaving dominant males, or sexually-receptive females, respectively. Our focus here was on neural responses to these complex odors because we know they induce behavioral and physiological responses in receiver males in social contexts^33,35^. The chemical identity of compounds released by *A*. *burtoni* in social contexts is unknown, however, so we chose examples of a sulphated sex-steroid and the amino acid alanine (both previously shown to be detected by the *A*. *burtoni* olfactory epithelium^41,42^) to serve as pure odorants related to reproduction and food as comparisons. All recordings were performed in the same region across all individuals, and electrolytic lesions verified the recorded neurons were located primarily in the dorsal Vv (Fig. 1b). Some lesions were at the border of Vv and the overlying dorsal nucleus of the ventral telencephalon (Vd).

**Figure 2:**
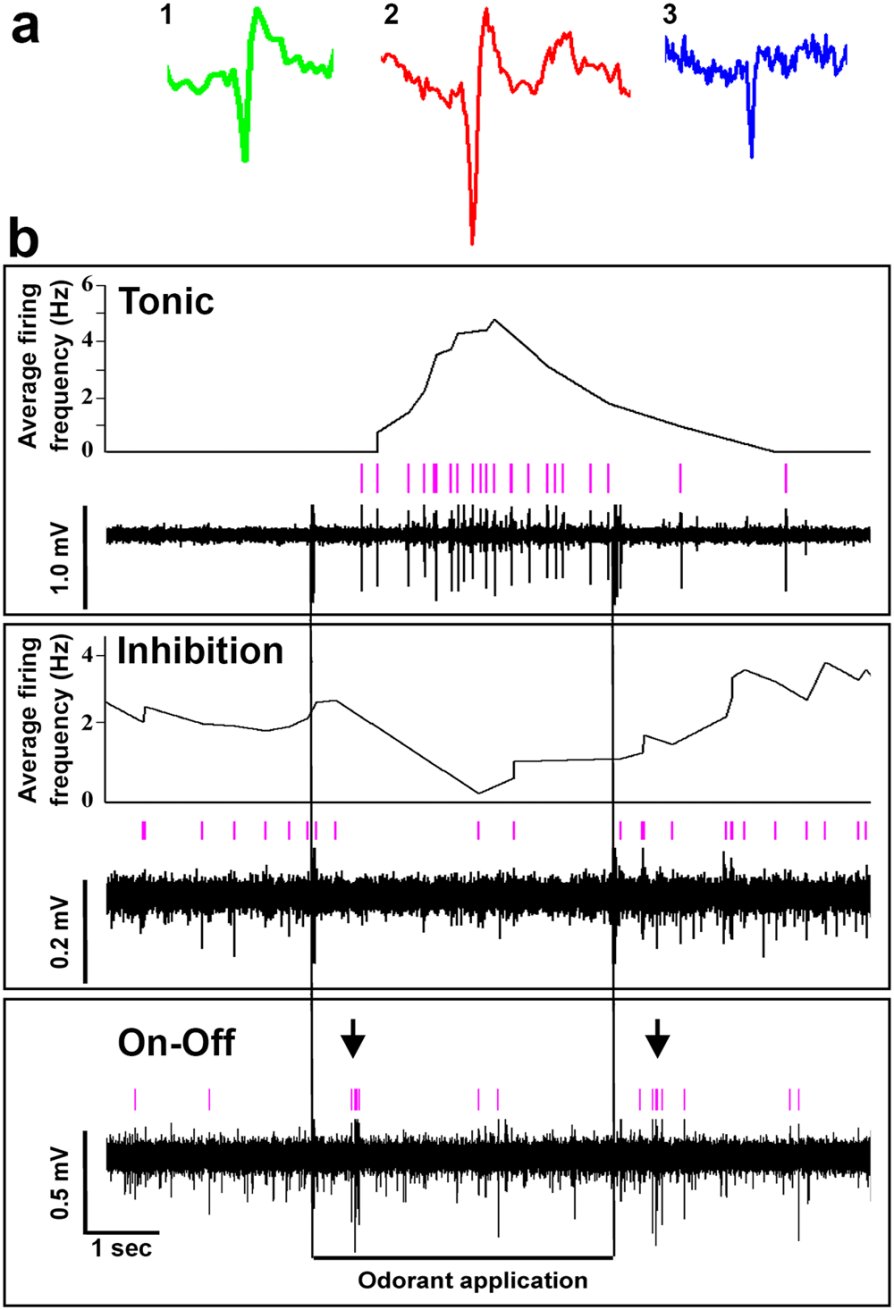
Several different odor-evoked spike response patterns were recorded from the ventral telencephalon in male *A*. *burtoni*. (**a**) Three different types of spike responses (Types 1–3) were recorded in all animals, characterized by distinct action potential shapes, durations, and amplitudes. Representative examples of each type are shown. (**b**) Several neural firing patterns were also recorded in response to test odors: tonic, inhibitory, on-off, and phasic-tonic. Representative examples are shown. Spike-sorted single-unit events (pink vertical marks) are shown above each recording trace. Tonic: dominant male, sex-steroid; inhibition: subordinate male, sex-steroid; On-off: subordinate male, female water (on and off bursts marked with arrows). See Fig. 3 for phasic-tonic examples (e.g. dominant male, female water odor; subordinate male, male water odor).

**Figure 3:**
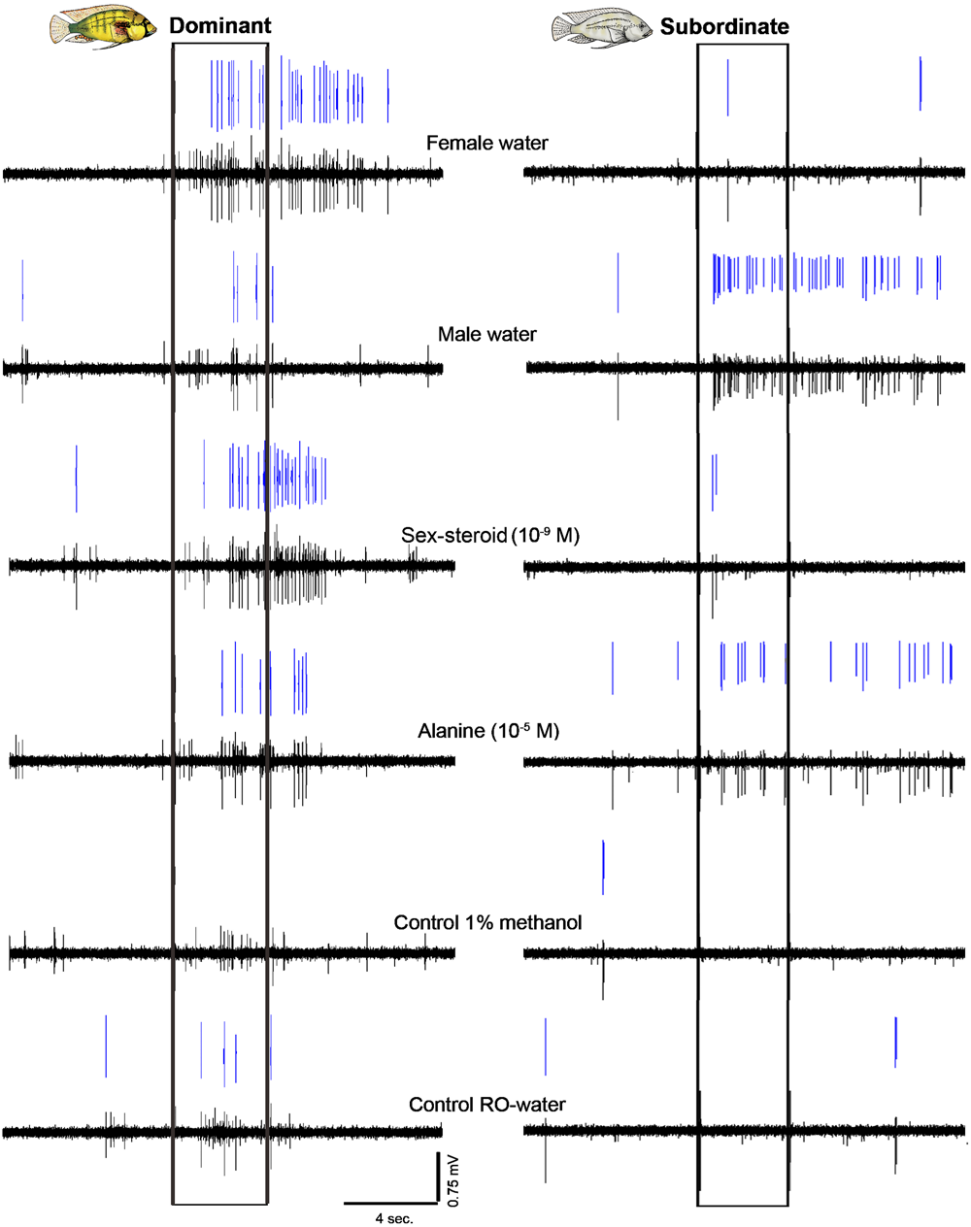
Neurons in the ventral telencephalon show different odor-specific excitatory responses in dominant compared to subordinate males. Representative multi-unit recording traces of ventral telencephalic neurons in a dominant and a subordinate *A*. *burtoni* male across all tested odors are shown. Notice that the neuron in the dominant male responds with an increase in spike rate when all four test odors are applied to the olfactory epithelium, with a robust response to the female water and sex-steroid. In contrast, the neuron in the subordinate male responds only to male water and alanine, but the response to male water is greater than that seen in the dominant male. For each odor, bottom shows the original recorded waveform trace and top (blue) shows the spike-sorted events of a single unit discriminated based on action potential shape.

Forebrain olfactory-sensitive neurons had slow spontaneous firing rates (range, 0.15–3.5 Hz) that did not differ between dominant (1.50 ± 1.81 s.d.) and subordinate (1.13 ± 0.67 s.d.) males (Student’s t-test, t = −0.91, P = 0.372). We identified three different types of responses from our multiunit recordings based on differences in action potential amplitude, duration, and shape: Type 1, amplitude = 0.30–0.65 mV, duration = 2.8–3.6 ms with short hyperpolarization of ~6.0 ms; Type 2, amplitude = 0.6–1.0 mV, duration = 5.6–8.0 ms with long hyperpolarization of 10–30 ms; Type 3, amplitude = 0.1–0.3 mV, duration = 1.6–2.0 ms with no hyperpolarization (Fig. 2a). These response types were encountered in both dominant and subordinate individuals during application of all of the test odors, and may represent different cell types (e.g. Type 1, principal/projection neurons; Type 2, modulatory neurons; Type 3, interneurons) similar to that described in the olfactory circuitry and decision centers of mammals^43,44^. We focused our analysis on Type 1 responses because they likely represent principal cells that provide output to other brain regions. Because there is a paucity of studies on neuron firing characteristics in this region in other fishes for any comparison, our classification of these responses as originating from principal/projection neurons is based on the fact that their spike firing properties (e.g. low baseline firing rate, interspike intervals >100 ms, action potential waveform shape, coincident firing with peak LFP response) are similar to those of principal cells in putative homologous striatal regions in mammals^44^. Neurons in both dominant and subordinate males also responded to test odors with several types of firing patterns: tonic, phasic-tonic, inhibition, and on-off (Figs 2b and 3). While our analysis is focused on Type 1 excitatory responses and it was not our goal to fully characterize these different responses here, we did observe inhibitory responses primarily in subordinate males after application of the sex-steroid, and to different stimuli in Type 3 cells of both social states. We were only able to record these inhibitory responses serendipitously in our multi-unit recordings, and while inhibitory neurons are important for neural circuit function throughout the brain, we did not have enough examples of inhibition to quantify their importance in olfactory processing here in Vv.

Excitatory Type 1 responses typically showed an increase in spike rate during the stimulus application period, and in many cases the response lasted for several seconds beyond the stimulus offset (see Figs 2b and 3). The onset latency of the increased spike rate response after application appeared faster for the two complex odors, male water and female water (≤0.3 sec), compared to the pure sex-steroid and alanine odorants (~0.5–0.8 sec). Odor-evoked spike responses from neurons in *A*. *burtoni* males were similar to that described in the forebrain of the catfish^45,46^. In the catfish forebrain, individual neurons responded to odorant classes that serve a similar behavioral function (e.g. amino acids and nucleotides for feeding), but not of food-related (amino acids, nucleotides) and socially-relevant odorants (bile salts)^45^. In contrast to the catfish, however, we observed individual neurons in the forebrain that responded to both amino acids and socially-related odor classes (sex-steroid, female water) (Fig. 3). This difference may be explained by the fact that our recordings were done in Vv, an area not sampled in the catfish. Neurons in the Vv of zebrafish also respond to many odorant types^39^, and receive biased inputs from specific glomerular clusters in the olfactory bulbs^7^. Using Ca^2+^-imaging and recording techniques in zebrafish, Yaksi *et al*. (2009) demonstrate that Vv neurons pool convergent mitral cell inputs to form overlapping odor representations, resulting in broad tuning (i.e. single Vv neurons respond to many different odorant types). Further, neurons in the zebrafish Vv responded more strongly to mixtures of odorants rather than their individual components^39^. The teleost Vv (putative homolog to some septal and striatal regions in mammals) is shared between the conserved social behavior network and mesolimbic reward pathways, suggesting it may be involved in regulation of general behavioral states associated with olfactory inputs.

### Odor-Evoked Spike Responses in the Ventral Telencephalon Differ with Male Social Status

**Figure 4:**
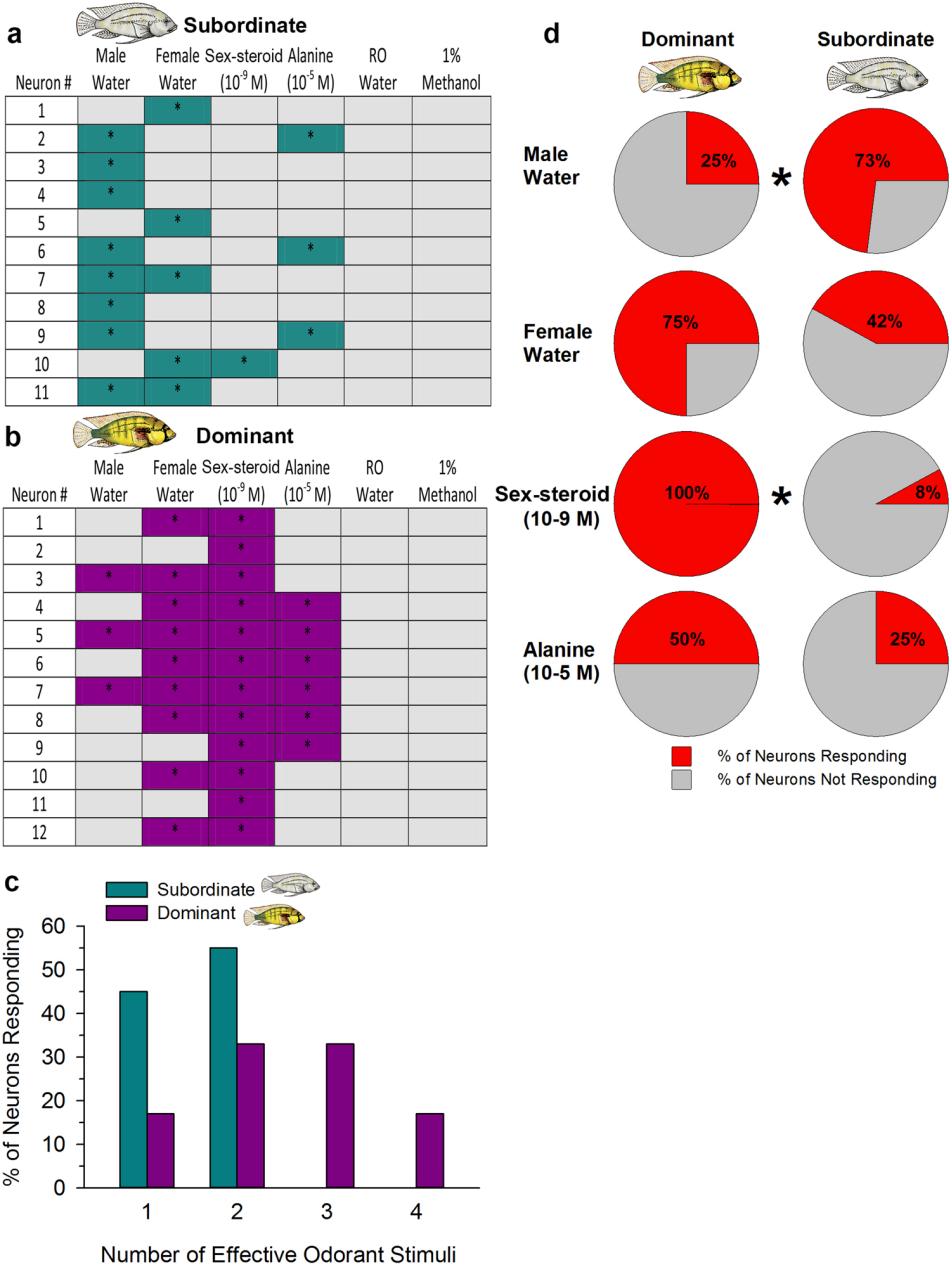
Odor-evoked excitatory responses of neurons in the ventral telencephalon differ with male social status in *A*. *burtoni*. Response properties for all individual recorded neurons in subordinate (**a**) and dominant (**b**) males to each tested odor are shown. Colored cells with asterisks represent excitatory responses based on criterion (see methods), while gray-shaded cells indicate no response. (**c**) The percentage of neurons responding to one, two, three, or all four of the tested odors in dominant and subordinate males. Neurons in subordinate males only responded to one or two of the tested odors, while the majority of neurons in dominant males responded to two or more odors. (**d**) The percentage of neurons in dominant and subordinate males that responded to each of the different tested odors also differed. A greater percentage of neurons in subordinate males responded to male water, while a greater percentage of neurons in dominant males responded to the sex-steroid, female water, and alanine. Asterisks indicate statistical differences from Fisher Exact tests at P < 0.05.

To examine odor-evoked spike responses in dominant and subordinate males, we first determined whether or not each recorded neuron showed an excitatory response to each test substance (Fig. 4). An excitatory spike response means that the synaptic inputs were sufficient to reach threshold and generate an action potential, which reflects neuronal output. There was no response based on our criterion (spike rate during odor application of 2 standard deviations above spike rate during pre-stimulus period) to application of the control RO-water or 1% methanol in any of the recorded neurons (Fig. 4a,b). To compare the odor-evoked response properties between dominant and subordinate males, we determined the percentage of recorded neurons that responded to one, two, three, or all four of the test stimuli. Neurons in subordinate males only responded to one (45%) or two (55%) tested odors, with none responding to more than two odors. In contrast, the majority (83%) of neurons in dominant males responded to two or more odors (2: 33%; 3: 33%; 4: 17%) (Fig. 4c). Thus, individual Vv neurons responded to an average of 2.5 odors in dominant males compared to 1.5 in subordinate males. To examine whether there were differences between dominant and subordinate males in the type of odor stimulus that evoked responses, we determined the percentage of neurons responding to male water, female water, sex-steroid, and alanine (Fig. 4d). While there were examples of neurons in both dominant and subordinate males that responded to all tested odor types, there was a greater percentage of neurons that responded to male water in subordinate males compared to dominant males (Fisher’s Exact test, P = 0.039). There was also a greater percentage of neurons in dominant males compared to subordinate males that responded to the sex-steroid (Fisher’s Exact test, P < 0.001; only 1 neuron in subordinate males showed a response). While there was approximately twice as many neurons in dominant males that responded to female water and alanine compared to subordinate males, it was not statistically different between the social states (FW, P = 0.10; Ala, P = 0.40). In sum, individual ventral telencephalic neurons in dominant males responded to several different odor types and more neurons were responsive to putative sex- (female water, sex-steroid) and food- (alanine) related odors compared to subordinate males. Further, more neurons in subordinate males compared to dominant males responded to odors released by other males.

### Odor-Evoked Spike Rates and Response Durations are Similar in Dominant and Subordinate Males

**Figure 5:**
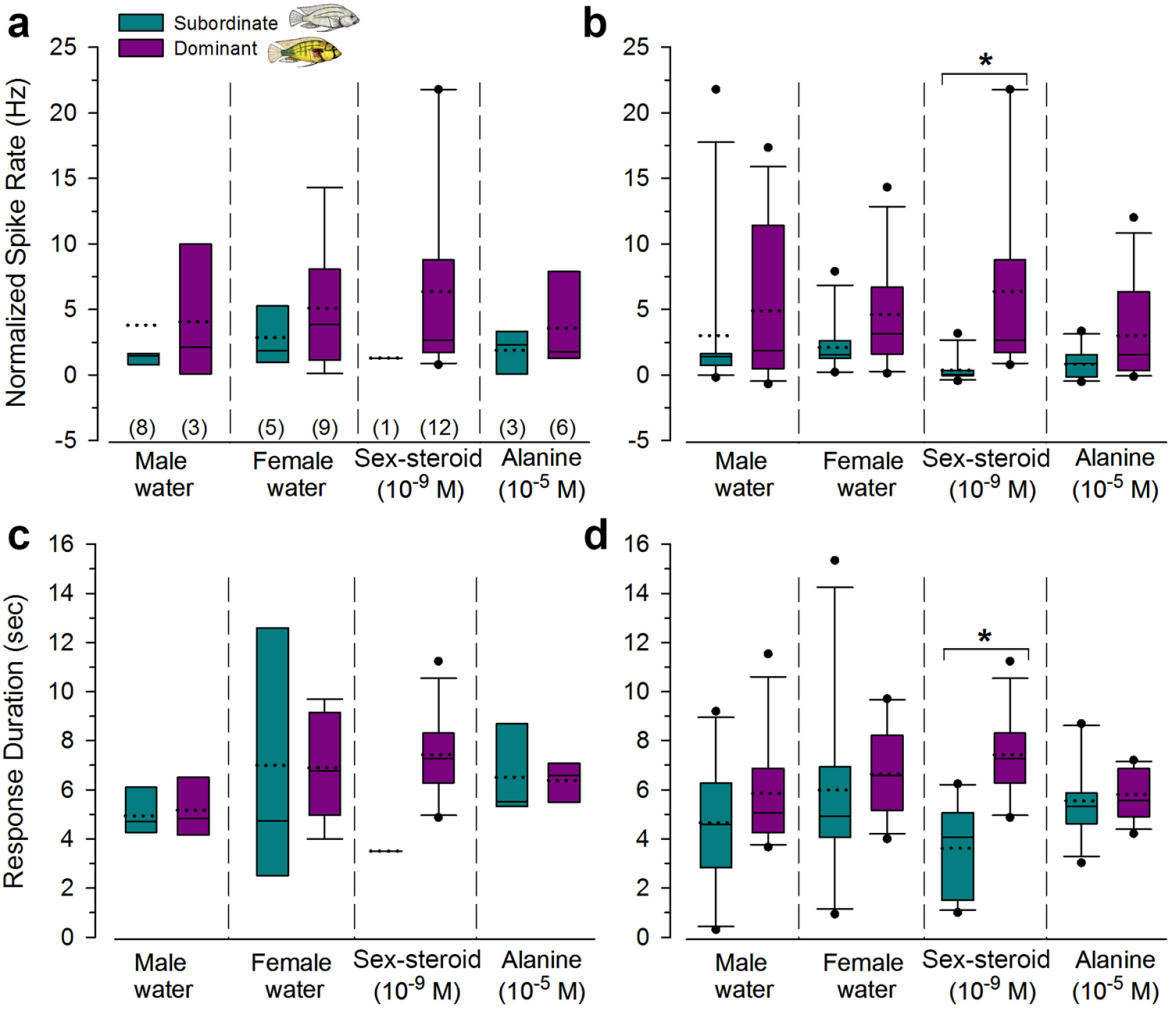
Odor-evoked spike rates and response durations of neurons in the ventral telencephalon are similar in dominant and subordinate *A*. *burtoni* males. Normalized spike rates during the stimulus application across different odor stimuli for only those neurons that showed an excitatory spike response based on criterion (**a**) and all recorded neurons together (**b**). Response spike rates were similar between social states, but were higher in dominant males only for the sex-steroid application. Response duration evoked from different odor stimuli for all recorded neurons (**c**) and only those neurons showing an excitatory response (**d**). Duration of the response was also longer in dominant males compared to subordinate males only for the sex-steroid. Boxes extend to the furthest points within the 25^th^ and 75^th^ percentiles and whiskers to the 10^th^ and 90^th^ percentiles. The median is represented as a solid line, mean as a dotted line, and data points outside the 10^th^–90^th^ percentile are dots. For B and D, N = 11 neurons, 7 fish for subordinate and 12 neurons, 7 fish for dominant. Sample sizes for responding neurons plotted in A and C are indicated in parentheses in A.

Because neurons in dominant males responded to several of the test stimuli, we next asked whether there were differences in the normalized evoked spike rates (spikes per sec., Hz) and response durations among odors and between social states. Odor-evoked spike rates were similar between dominant and subordinate males, as well as among different test odors within each social state. This was true when we compared only those neurons that responded based on the criteria, as well as all the recorded neurons collectively. The one exception was that spike rate was higher in dominant males compared to subordinate males but only for the sex-steroid when all neurons were included in the analysis (GLMM, stimulus x status: F_5,29_ = 3.35, P = 0.016) (Fig. 5a). Similarly, response duration to the sex-steroid was also longer in dominant males compared to subordinate males for all neurons tested (stimulus x status: F_5,37_ = 2.87, p = 0.027), but there were no other differences among test odors or between social states (P > 0.05) (Fig. 5b). This is not surprising however, because we only encountered one neuron in a subordinate male that showed an excitatory response to the sulphated sex-steroid, while 100% of the neurons in dominant males showed an excitatory response (thus statistical tests could not be performed on only responding neurons). Thus, while the proportion of neurons that responded to each odor type differed with male social status, the spike firing characteristics of neurons that did respond were similar in dominant and subordinates. Our odor-evoked spike characteristics suggest that we are recording from principal/projection neurons that provide output information. Because odor-evoked spike rates are similar in dominant and subordinate males, it suggests that whether or not a neuron reaches threshold and generates an action potential (e.g. percentage of responsive neurons) is more important than spike rates for linking biologically-relevant olfactory information received by the ventral telencephalon to other brain areas involved in decisions related to behavioral outcomes.

### Local Field Potentials Reveal Differences in Olfactory-Mediated Neural Processing Associated with Male Social Status

**Figure 6:**
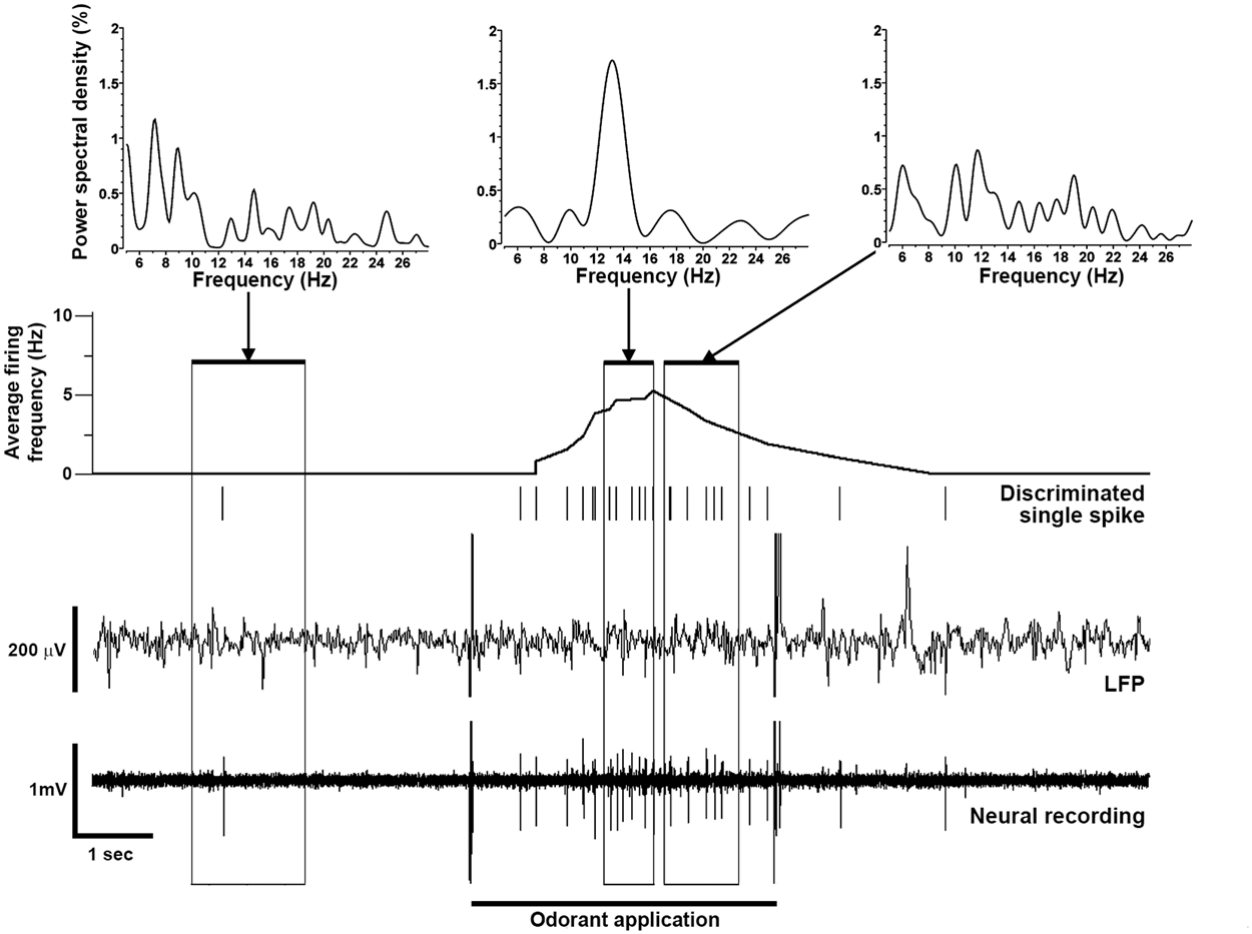
Power spectral densities of local field potentials (LFPs) showed prominent peaks during odor application that is indicative of complex processing. Example of simultaneous LFP and multi-unit spike recording traces from a dominant male exposed to female water (stimulus artifacts are visible at the start and stop of application). A discriminated single-unit spike (vertical marks) and average firing frequency (Hz) is shown above the raw recording traces. Power spectral densities (top) are shown for the indicated times before and during the odor application. Note a prominent spectral peak at ~12–15 Hz that occurs ~2 sec after odor application, but is absent before and after this time period.

**Figure 7:**
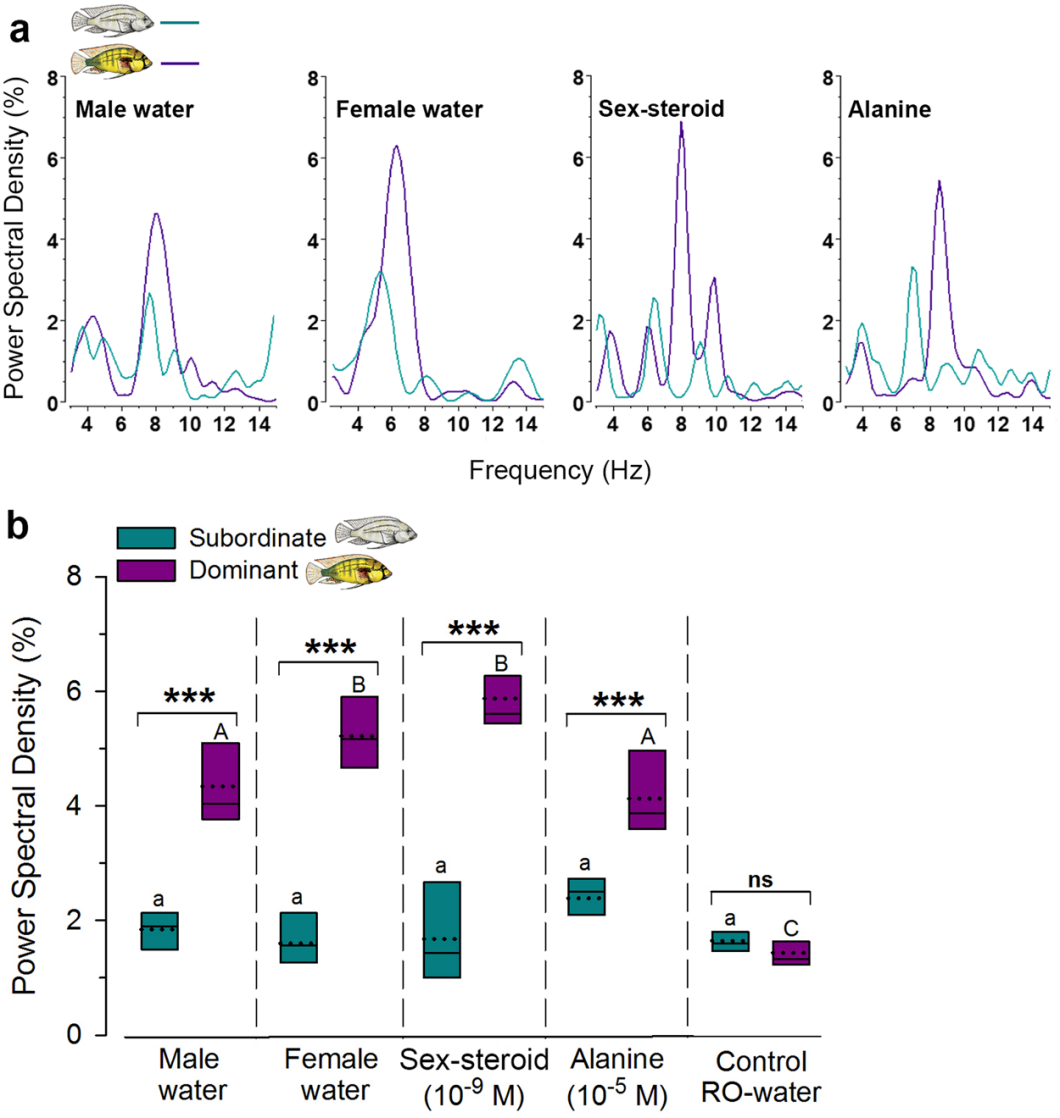
Odor-evoked LFP power spectral densities in the ventral telencephalon differ with male social status. (**a**) Representative power spectral densities after application of each odor type in a dominant and a subordinate male. Y-axis is the percentage of spectral power of frequencies between 3 and 50 Hz computed over 1.5 sec. of response. Note the greater spectral power density peaks in the 4–9 Hz range in dominant (>4%) compared to subordinate (<3%) males for all odors. (**b**) Quantification of odor-evoked power spectral densities in the theta frequency range (4–9 Hz) show that dominant males had spectral densities 2-3-fold greater than subordinate males for all tested odors, while control RO-water application did not differ. Power spectra also differed across different odors in dominant males, but not in subordinate males, with female water and sex-steroid showing greater LFPs than male water and alanine. See Fig. 5 for box plot description. Lines with asterisks (***) indicate differences between social states within each odor at P < 0.001. ns, not significant. Different letters indicate differences at P < 0.05 between odors within each social state (lowercase, subordinate; uppercase, dominant). N = 7 neurons for each social status.

Recordings of single- and multi-unit firing revealed that more neurons in the ventral telencephalon of dominant males compared to subordinate males send output to other neurons and potentially other brain regions, so we next evaluated LFPs to test whether synaptic inputs to this region also differed between social states. Importantly, these inputs may represent incoming information collectively from both the olfactory system (e.g. epithelium and bulb) and from synaptic inputs originating in other brain regions (e.g. modulatory). To examine this non-spike-related processing, we compared power spectral densities of the LFP recordings during odor application between dominant and subordinate males. LFPs result from a combination of synaptic activity within ~100–250 µm of the recording electrode and provide useful temporal information on the collective activity of groups of neurons involved in complex decisions^47–49^. Further, in contrast to spike activity which reflects neuronal output, LFPs are thought to primarily reflect the dendritic/synaptic activity of neuronal inputs, and are a fundamental characteristic of large neuronal networks processing information related to behavioral and perceptual states^47,50^. LFPs are transient oscillations, and analysis of power spectral densities in *A*. *burtoni* showed absence of a clear spectral peak before odor application (Fig. 6). In contrast, prominent spectral peaks in theta (4–9 Hz) or low beta (10–15 Hz) frequency ranges occurred during odor application that were coincident with neuron firing, primarily in dominant males (Fig. 6). There was a significant interaction between social status and the type of odor present in terms of LFP theta (4–9 Hz) power spectral densities (2-way RM ANOVA, status x odor: F_4,69_ = 22.54, P < 0.001), illustrating that LFP differences depended on social status (Fig. 7). Power spectral densities of theta oscillations were 2–3 fold higher in dominant males compared to subordinate males for all tested odors (status: F_1,69_ = 145.73, P < 0.001; Holm-Sidak, P < 0.001), while exposure to control RO-water did not differ between social states (P = 0.594) (Fig. 7b). Theta oscillations also differed among different odor types (odor: F_4,69_ = 40.273, P < 0.001), but only in dominant males (P < 0.05), and not in subordinate males (P > 0.05). In dominant males, spectral densities were greater for female water and sex-steroid applications compared to both male water and alanine (P < 0.05), and all tested odors were greater than the control (P < 0.001) (Fig. 7b). In contrast to theta waves, there were no differences between dominant and subordinate males in low beta (10–15 Hz) (2-way RM ANOVA, status: F_1,69_ = 1.155, P = 0.324; status x odor: F_4,69_ = 1.017, P = 0.418) or high beta (16–28 Hz) (status: F_1,69_ = 0.298, P = 0.605; status x odor: F_4,69_ = 0.741, P = 0.574) oscillations. LFP analysis further demonstrates that the odor-evoked neural state of Vv in dominant males differs from that in subordinate males, likely reflecting greater synaptic inputs either from the olfactory system or from other brain regions.

### Electro-olfactogram (EOG) Recordings Reveal Status-Dependent Differences in Responses to Behaviorally-Relevant Odors

**Figure 8:**
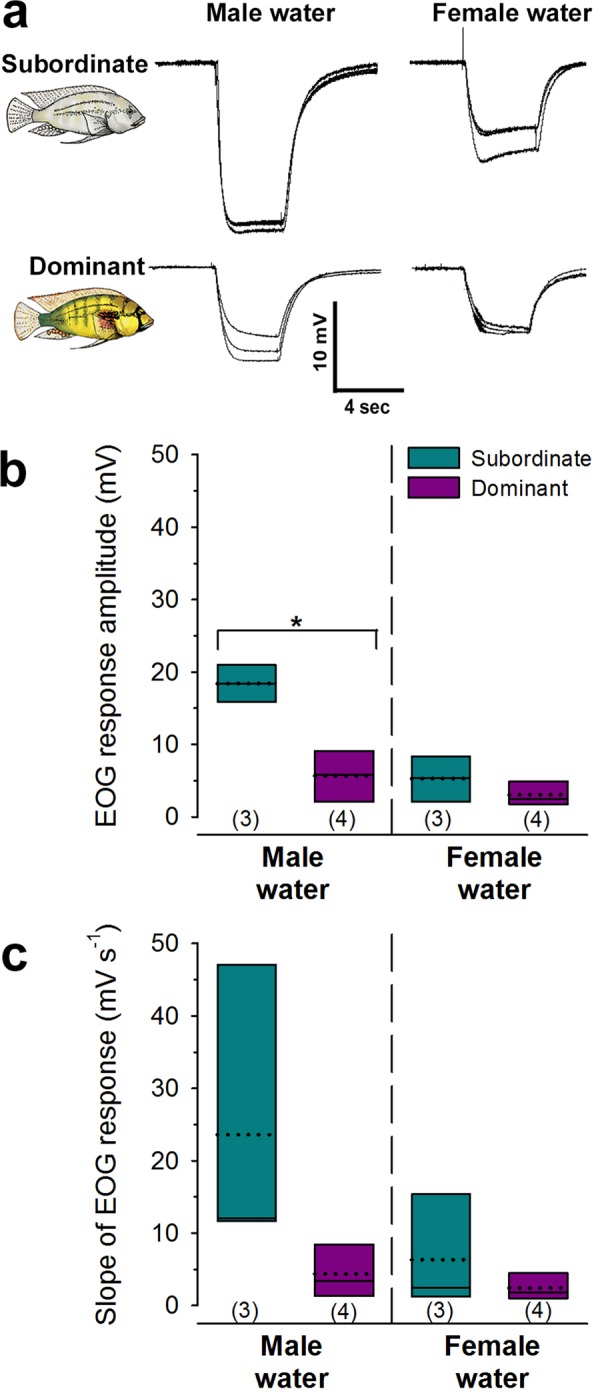
Odor-evoked electro-olfactogram responses differ with male social status. (**a**) Example of three overlaid EOG traces each from an individual subordinate and dominant male to illustrate the stability and repeatability of the responses to repeated stimulus applications of male-conditioned and female-conditioned water. (**b**) EOG amplitudes in response to male water were greater in subordinate males, while responses to female water did not differ between the social states. (**c**) The slope of the EOG responses did not differ between dominant and subordinate males for either male water or female water odors. See Fig. 5 for box plot descriptions. Line with asterisk indicates a statistical difference between social states at P < 0.05. Sample sizes (number of fish) are shown in parentheses.

Our LFP recordings revealed status-dependent differences in the synaptic inputs to Vv, but they do not distinguish whether these inputs originate from the olfactory system itself or from other brain processing regions. To examine this further, we used electro-olfactogram recordings in dominant and subordinate males as in our previous study^42^ to test whether there were inherent differences at the olfactory epithelium in response to complex male-conditioned and female-conditioned water odors (Fig. 8a). Our focus was on these complex odors because we know that these mixtures released from conspecifics induce behavioral responses in male receivers during social interactions, and the multitude of odorant compounds likely contained in the samples has high probability of interacting with many different types of olfactory receptor neurons to produce a response. In contrast, the biological relevance of the tested sulphated sex-steroid is not known, and we were unable to compare EOG characteristics to the sex-steroid because responses in both dominant and subordinate males were not reliably distinguishable from responses to control water solutions. The fact that we recorded reliable responses to the sex-steroid in Vv neurons highlights the limitation that EOGs are not a true measure of olfactory information transmitted to the brain (i.e. EOGs are summed potentials reflecting activity of both sensory and non-sensory portions of the olfactory epithelium and are not equivalent to action potentials transmitted from ORNs to the brain). EOG amplitudes in response to the male water were greater in subordinate males compared to dominant males (GLMM, odor x status, F_1,4_ = 9.028, p = 0.040) (Fig. 8b). While the slope of the EOG response also appeared greater in subordinate males compared to dominant males, it was not statistically significant (GLMM, odor x status, F_1,4_ = 2.940, p = 0.162) (Fig. 8c). EOG response amplitude and slope did not differ between male social states for the female-conditioned water (P > 0.05). Thus, the olfactory epithelium is more responsive to male-conditioned water in subordinate males compared to dominant males suggesting shifts in sensitivity at the epithelium to specific compounds within the mixture and potentially greater olfactory inputs to processing centers such as Vv specifically for this complex odor.

## Discussion

We reveal here that responses of olfactory-sensitive neurons in Vv of the ventral telencephalon of a cichlid fish differ with male social rank. This discovery, to our knowledge, is the first instance of odor-evoked differences in neuron response properties in a higher processing-center of the brain associated with male dominance status in any vertebrate. This suggests that olfactory processing in conserved decision centers may be fundamentally different depending on the animal’s physiological state that is tied to their social rank. Combined analysis of LFPs and action potentials also indicate that both synaptic inputs and odor-evoked outputs of the Vv differ between states. While little is known about the role of Vv in olfactory-mediated behaviors in fishes, homologous septal and striatal nuclei in mammals receive projections from the olfactory bulbs and are involved in linking socio-sexual olfactory-related signals with contextual rewards^51,52^. These studies, coupled with our results of social status differences in Vv neurons further support an important and conserved role for this nucleus in olfactory-mediated social behaviors across vertebrates. This olfactory plasticity would be advantageous for any species that relies on olfaction to mediate lifestyle-related behaviors such as reproduction, territoriality, and feeding that may fluctuate with social rank.

Neurons in the ventral telencephalon of dominant male *A*. *burtoni* appear more broadly tuned to several odors compared to subordinate males. However, it should be noted that much of this putative broad tuning is attributed to the high response of neurons to the sex-steroid in dominant compared to subordinate males. In contrast, neurons in subordinate males typically showed an inhibitory response to the sex-steroid, but because our focus was on excitatory responses, the significance of this inhibition deserves future attention. While one interpretation is that Vv neurons in dominant males are detecting a wider variety of compounds, other interpretations are that more neurons are increasing their sensitivity to this particular (and potentially other) compound in dominant males, or neurons in subordinate males lose or decrease their sensitivity (i.e. shifts in sensitivity). Since the *A*. *burtoni* olfactory system detects many types of conjugated steroids^41^, additional forebrain recordings that test responses to more odorants are needed to address these possibilities.

**Figure 9:**
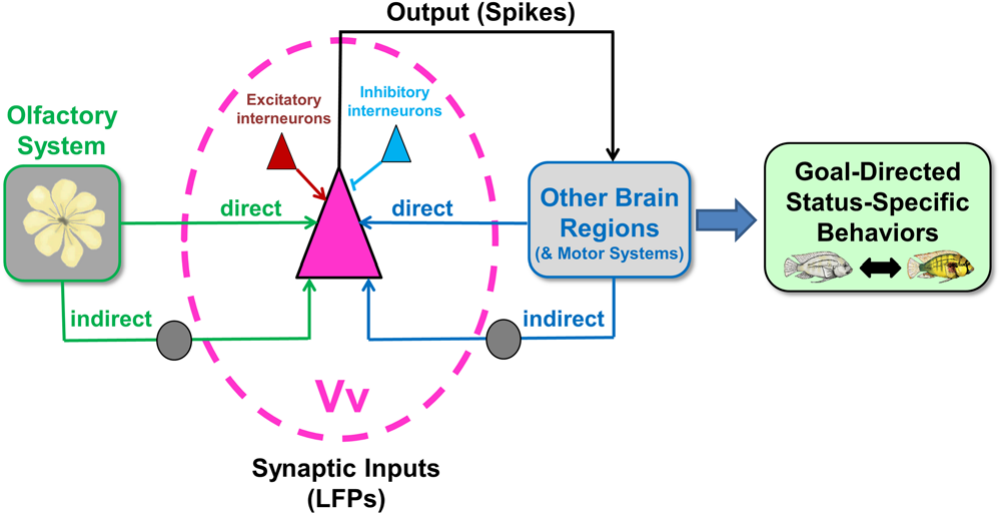
Schematic of proposed neural processing involved in olfactory-mediated status-specific behaviors in the cichlid. Simplified schematic diagram illustrates the relevant synaptic inputs to the olfactory-responsive output neurons that were recorded in the ventral telencephalon (Vv). Vv receives direct (extrabulbar) and indirect (via olfactory bulb) inputs from the olfactory epithelium, as well as direct and indirect inputs from other brain regions. Within Vv, there are also various excitatory and inhibitory (e.g. GABAergic) inputs that contribute to the LFP recordings, which collectively result in output spikes eventually leading to goal-directed and status-specific behavioral responses in the subordinate and dominant male phenotypes.

What might account for these social status differences in olfactory forebrain responsiveness? There are several possible reasons, none of which are mutually exclusive, and a simplified summary schematic of the inputs and outputs of Vv relevant to this discussion is shown in Fig. 9. First, there may be differences in morphology or sensitivity at the level of the olfactory epithelium (e.g. ORN abundance, neuromodulation)^53^, providing differential inputs to Vv between social states. In support of this possibility, our EOG recordings demonstrate differences in responses between dominant and subordinate males specifically to male water odors. Further, our previous EOG recordings revealed greater response amplitudes and steeper slopes to amino acid stimuli in dominant compared to subordinate males^42^, illustrating plasticity in the epithelium that is consistent with different inputs to Vv coming from the olfactory system itself. This may mediate important behavioral decisions based on reception of context-specific olfactory information salient to each social state. It is relevant to note, however, that EOG recordings are summed potentials reflecting activity of the entire epithelium (sensory and non-sensory) and are not equivalent to action potentials transmitted from ORNs to the brain; response properties of ORNs should be examined in future studies and it is possible that sensitivity to specific compounds is gained or lost in different social states. A study in goldfish also revealed that sex-steroids influenced olfactory sensitivity (measured as EOG responses) and courtship behaviors on different timescales^54^, highlighting the possibility that effects at the olfactory periphery can occur via independent mechanisms from those in the brain. Thus, both peripheral and central plasticity of olfactory abilities could account for changes in neuron responses in the service of social behaviors.

Second, there may be different inputs or changes in neural circuitry between Vv and other brain regions between social states, which is supported by our results showing greater LFP spectral densities in dominant males. This plasticity with social status may alter the synaptic connections or relative excitatory versus inhibitory activity involved in processing that leads to olfactory-mediated behavioral decisions. The target Vv recording region in *A*. *burtoni* has both glutamatergic (e.g. expresses *vglut2*.*1*) and GABAergic (e.g. expresses *gad1* and *gad2*) neurons that could be involved in local circuit modulation^55^. Interestingly, a recent study demonstrated that a change in the relative weight of excitatory and inhibitory inputs to specific neurons in the brain alters pheromone-related behavioral outcomes in *Drosophila* species^56^. A similar mechanism may exist here in the vertebrate brain where the circuitry reversibly shifts from being balanced in favor of submissive behaviors in subordinates to a balance in favor of reproduction and aggression in dominants. This type of circuit plasticity is particularly relevant in male *A*. *burtoni* that are well-known for their ability to rapidly (minutes to days) change neuron morphology, gene expression, behavior, and physiology during status transitions^57^.

Third, olfactory responsiveness in Vv could differ between dominant and subordinate males due to neuromodulation. Central modulation of sensory perception by sex-steroids, neuropeptides, or biogenic amines is well-documented across vertebrates^20,58–61^. Vv/Vd and the olfactory bulbs of *A*. *burtoni* express many different neuromodulators and their receptors (e.g. dopamine, serotonin, NPY, AVT, GnRH, CART, AgRP, and others)^62–65^. Further, dominant males have greater levels of some sex-steroid receptor subtypes in both Vv and the olfactory bulbs compared to subordinate males^66,67^. These receptor changes provide a neural substrate for modulation at multiple sites along the olfactory pathway, which could also account for the greater LFP spectral densities reflective of increased synaptic processing in dominant males. Future studies, including recordings in males transitioning between subordinate and dominant states, are needed to determine the exact mechanism(s) involved in this status-dependent olfactory plasticity of the cichlid ventral telencephalon.

Neurons in Vv of dominant males were overall more responsive to putative sex- (female water, sulphated sex-steroid) and food-related (alanine) odors compared to subordinate males. In contrast, subordinates were specifically more responsive to male-released odors. These differences likely facilitate the olfactory-related needs in males to match their status-specific lifestyles. For example, dominant males are focused on courtship, spawning, and territory defense, leaving less time to feed. We know that odors released from gravid receptive females increases searching and courtship behaviors in dominant males, demonstrating the importance of olfactory signals for reproduction^34,35^. Because these reproductive and territorial behaviors (and their physiological consequences) are energetically costly, the greater responsiveness of neurons in Vv, as well as the olfactory epithelium^42^, to food-related odorants (amino acids) may maximize prey/food detection and capture to maintain energetic balance.

Subordinate males on the other hand have limited reproductive potential but more feeding opportunities to invest in growth in anticipation of acquiring a territory and rising in rank^68^. High responsiveness to male-released odors in these subordinate males (revealed in EOGs and Vv spike recordings) would allow them to monitor the dominance status of other males in the population, providing advantages for non-contact social assessment. Because LFP spectral density is lower in Vv of subordinate males, the circuitry may require greater olfactory inputs from the epithelium to drive neural outputs needed to generate goal-directed behaviors. Subordinate males do not hold territories and therefore have limited mating opportunities, so the ability to quickly locate vacant territories or selectively challenge only those dominant males that they have a greater chance of defeating improves their reproductive fitness. In the related tilapia (*Oreochromis spp*.), for example, dominance status of individuals is conveyed via chemicals released in their urine and reduces aggression in rivals^12,69,70^. It is also possible that substances released from dominant males function to keep subordinate males behaviorally and physiologically suppressed^32^. Previous work demonstrated that visual signals from dominant males alone are not sufficient to physiologically suppress subordinate males, suggesting other senses like olfaction may be involved^32^ but this requires further experimental testing. Importantly, however, subordinate males can still detect all odors to continuously monitor their environments, but reduced LFP activity suggests that the information is not salient to generate rapid context-appropriate behavioral decisions as it is in dominant males. Thus, the observed status-dependent olfactory plasticity observed here in the forebrain matches the lifestyle trade-offs between reproduction and feeding/energetics in males of this species. Because dominance hierarchies are widespread across the animal kingdom, these types of status-dependent trade-offs are common. Olfactory plasticity in the brain may be a conserved mechanism allowing species to gate sensory information in different social states to optimize survival and reproductive fitness.

We also identify differences in odor-evoked LFPs between dominant and subordinate male *A*. *burtoni*, further supporting inherent status-dependent processing within the Vv in this species. The use of LFP oscillations to understand complex neural computations, including olfactory-related processing, is commonplace in mammals but rarely utilized in other vertebrates^47,71,72^. LFPs typically reflect intrinsic biophysical and synaptic features of the system within a species, are a hallmark of the olfactory system across phyla, and can be influenced by neuromodulators^71^. Because LFPs reflect neuronal activity within ~100–250 µm of the recording electrode, transient fluctuations in the peak spectral densities primarily represent aspects of changing synaptic inputs that are not revealed by solely measuring neuron spikes^48,72^. In fact, while male cichlids in our study showed clear differences in the proportion of neurons that responded to different odors (e.g. reached threshold to generate an action potential output), there were no differences in the firing characteristics (rate and duration) between social states, but LFP spectral densities were 2–3 fold higher in dominant males. The transient rhythmicity of LFPs helps link activity within and across brain areas, particularly during on-going and goal-directed behaviors, and is involved in decision-making, reinforcement-based learning, and integrating anatomically distributed processing in the brain^44,73,74^. A recent study in primates also demonstrated that LFPs are a stronger predictor of imminent behaviors than neural spikes^75^. Thus, the greater spectral densities within the theta frequency range of dominant males indicate that more incoming synaptic information is used to generate and compute outputs to other circuit components in the service of initiating an appropriate context-dependent behavioral response.

While the neuronal mechanisms responsible for LFPs in *A*. *burtoni* require further study, our results suggest that increased and synchronized synaptic activity may drive odor decision processing in high-ranking individuals to produce the suite of ~17 stereotypical dominance behaviors^76,77^. This matches the lifestyle of dominant males that use olfactory information for rapid decisions related to conflicting behaviors such as fighting, courting, or eating. Theta oscillations in the mammalian olfactory brain are typically associated with the respiratory cycle, which brings odors into the olfactory epithelium via sniffing^49^. However, there is also evidence that theta rhythms may coordinate and relay sensorimotor information within and across different sensory systems to mediate behaviors and serve ‘top-down’ modulating, ‘attentional’, and other context-defining functions^49,50,78^. Our LFP analyses here uncovering social status differences in the cichlid extends the utility of these rhythmic oscillations to fishes, by revealing important computational features of heterogeneous groups of neurons that share common inputs. LFPs may provide insights on function in the non-mammalian vertebrate brain, and future comparisons with mammals may divulge important conserved processing mechanisms.

In summary, we provide electrophysiological evidence from both spike responses and LFPs for social status differences in olfactory responsiveness of the fish ventral telencephalon. Sensory plasticity related to an animals’ physiological condition (e.g. hormonal, nutritional state) is documented across vertebrate taxa and for multiple senses. Our results reported here, however, are the first demonstration of social status-dependent plasticity in olfactory processing capabilities within the vertebrate forebrain. While *Astatotilapia burtoni* lives in a dynamic social environment in which transitions in male social rank are common and associated with changes in olfactory valence, this discovery also has important implications that reach far beyond fishes. Dominance hierarchies with frequent switches in social position are widespread in social animals, with examples from insects to humans^79,80^. Olfactory plasticity in the brain can modulate behavioral outputs to match activities of an individuals’ current rank or physiological state in many taxa. This flexibility may reflect evolutionary processes to optimize health, reproductive fitness, and survival in animals’ that experience fluctuations in social or other environmental conditions.

## Materials and Methods

### Animals

African cichlid fish *Astatotilapia burtoni* (Günther 1894) were laboratory-bred and originally derived from a population collected from Lake Tanganyika, Africa. Fish were maintained in mixed-sex groups in flow-through aquaria under conditions similar to what they experience in the lake (pH 8.0, 28–30 °C, 300–50 µS cm^−1^, 12 L:12 D light cycle, constant aeration). Fish were fed cichlid flakes daily (Aquadine, Healdsburg, CA, USA). All experiments were performed in accordance with the recommendations and guidelines provided by the National Institutes of Health Guide for the Care and Use of Laboratory Animals, 2011. The protocol was approved by the Institutional Animal Care and Use Committee (IACUC) at Louisiana State University, Baton Rouge, LA.

### Experimental Setup

Our goal was to compare *in vivo* response characteristics of olfactory-sensitive neurons in the ventral telencephalon of male cichlids that differed in social and reproductive state. Dominant and subordinate males were chosen based on their status-specific coloration and stereotypical behaviors, as in previous studies^42,81,82^, and reproductive investment was verified after the recording experiments by measuring gonadosomatic index (GSI = [gonad mass/body mass] * 100). Briefly, dominant males (standard length, SL = 5.78 ± 0.94 cm s.d.; body mass, BM = 4.47 ± 3.06 g s.d.; GSI = 0.71 ± 0.43) were selected based on presence of bright coloration and display of stereotypical territorial (lateral displays, border fights, frontal threats, chases) and reproductive (body quivers, leads, tail waggles, digging) behaviors. Subordinate males (SL = 5.67 ± 0.73 cm s.d.; BM = 3.88 ± 1.31 g s.d.; GSI = 0.27 ± 0.04) were chosen based on faded coloration (with no eye-bar) and display of submissive behaviors such as fleeing, hiding, and hovering at the water surface. Fish were netted from community tanks in the morning prior to feeding, placed in benzocaine anesthetic (0.05–0.1% dissolved in cichlid-system water), and immobilized with an intramuscular injection of the paralytic agent pancuronium bromide (2.5 µg g^−1^ BM in 0.9% NaCl). The fish was positioned in a Plexiglass holder, stabilized by orbital clamps, and kept moist by wet Kimwipes®. Ventilation was provided by a gravity-fed tube inserted into the mouth supplying a constant flow of aerated cichlid-system water [Reverse osmosis (RO) water supplemented with Tanganyika buffer (Seachem, Madison, GA, USA) to pH 8.0 and Cichlid Lake Salt (Seachem) to 300–400 µS cm^−1^] over the gills throughout the recording experiment. The water used during experiments was 22–25 °C, which is slightly cooler than their holding temperatures, but fish were acclimated to this temperature for 30–40 min. prior to experiments and experimental conditions were identical across all individual fish tested. The telencephalon was exposed by dorsal craniotomy and access to the olfactory epithelium was achieved by removal of a small amount of tissue surrounding the single naris opening on the left side of the fish.

### Recording Setup

Single and multiunit neuron activity was recorded extracellularly from the ventral telencephalon of dominant and subordinate males (Fig. 1a). Using stereotactical coordinates, the electrode (Parylene C-insulated Tungsten Microelectrodes; 2–5 MΩ impedance; A-M Systems, Carlsborg, WA, USA or gold and platinum-plated metal-filled glass micropipettes; <1 MΩ impedance) was advanced vertically into the telencephalon via a micromanipulator driven by a hydraulic microdrive. The target recording location was the ventral nucleus of the ventral telencephalon (Vv), which is homologous to both septal and striatal regions in the tetrapod brain. The ventral Vv is the putative teleost homolog of the mammalian lateral septum, while the dorsal Vv is homologous to the striatal external globus pallidus (although many forebrain teleost-mammalian homologs remain uncertain)^36,37,83^. The Vv was chosen because studies in other fishes identify it as an important olfactory processing region^5,7,39^, as well as a decision center shared by the social behavior network and mesolimbic reward pathways^40^. The Vv in fishes, including *A*. *burtoni* (unpublished observations), also receives extra-bulbar projections directly from the olfactory epithelium that may have evolved to mediate rapid pheromone-related behaviors in aquatic vertebrates^84–86^. The electrode was slowly advanced vertically by ~20–50 µm steps to a depth of ~1.5–2.0 mm from the brain surface. Once a spontaneously active unit was encountered, several of the test stimuli were applied to the ipsilateral olfactory epithelium. If the neuron responded with increased firing to at least one of the test stimuli, recording continued and the full complement of test substances were applied. If there was no response, the electrode was advanced further to locate a new neuron. This protocol was identical across individuals to minimize recording bias. Because of the low spontaneous firing activity of neurons in this region and the difficulty in finding and isolating olfactory-sensitive neurons, our focus was on neurons that responded with an increase in firing (excitatory). Neural responses were amplified (Grass Instruments P511k, Astro-Med Inc., West Warwick, RI, USA) and filtered (band-pass 30–3000 Hz), monitored visually (on computer) and acoustically (speaker), digitized on a CED Micro 1401 A-D converter running Spike 2 software (Cambridge Electronics Design, Inc., Cambridge, England), and stored on computer for later analysis.

Local field potentials (LFPs) were also simultaneously recorded with the same electrode used for single and multiunit recordings (Fig. 1a). LFP activity was amplified (Grass Instruments P-511), band-pass filtered (3–300 Hz), and digitized and stored as above onto a separate Spike 2 channel. LFPs are rhythmic oscillations found within many brain regions, and are a common characteristic of higher processing and cognitive tasks. They result from a combination of activity from action potentials and other membrane-related events such as synaptic activity, calcium spikes, intrinsic currents, spike after-polarizations, ephatic effects, and neuron-glia interactions^47^. LFPs provide useful temporal information on the collective behavior of groups of neurons within a radius of ~100–250 µm surrounding the electrode, are primarily reflective of synaptic/neuronal inputs to a region, and supply additional data on neural processing not obtained from solely measuring action potentials^48,87^. Recordings of LFPs are commonly used in mammals to reveal features related to learning and memory and other complex processing^47,88–90^, and oscillations in LFPs are a hallmark of olfactory processing across taxa^71^. While LFP oscillations are underutilized in fishes^91^, we show here that they can provide useful information for characterizing odor-evoked responses in higher processing centers, as well as how the intrinsic properties of the brain influence olfactory responses.

Our previous Electro-olfactogram (EOG) recordings from the olfactory epithelium revealed that dominant males had EOG responses to amino acids with greater peak amplitudes and steeper slopes compared to subordinate males, but socially-relevant odors were not tested^42^. To test whether inputs from the olfactory epithelium to our Vv recording region might differ with male social status, we used an identical EOG recording protocol^42^ in additional fish (Subordinate: SL = 6.24 ± 0.64 cm s.d.; BM = 5.00 ± 1.90 g s.d.; GSI = 0.38 ± 0.29; N = 3; Dominant: SL = 5.45 ± 0.29 cm s.d.; BM = 4.17 ± 0.66 g s.d.; GSI = 0.77 ± 0.10; N = 4). Every attempt was made to ensure EOG recordings were done without prior knowledge of fish status (one experimenter selected the fish and another performed the recordings), but this is difficult because dominant and subordinate males have different body coloration. Briefly, the olfactory epithelium was exposed and an Ag/AgCl pellet recording electrode (World Precision Instruments, Sarasota, FL.) fitted with a saline-agar-filled glass capillary tube was positioned immediately above the epithelium while a reference electrode was placed on the skin between the eyes. EOG responses were DC amplified (Grass P-18, Astro-Med, West Warwick, RI), digitized on a CED Micro 1401 A-D converter running Spike 2 software (Cambridge Electronics Design, Cambridge, UK), and stored on computer. Only EOG responses to the complex odors of female-conditioned water and male-conditioned water (see Odor Stimuli and Delivery section below) were compared here for analysis, and each test odor was presented 5–8 times in each fish.

At the end of all recording sessions, each fish was measured for standard length (SL), weighed for body mass (BM), and sacrificed by rapid cervical transection. Gonads were removed and weighed (gonad mass, GM) to calculate GSI as a measure of reproductive investment and to verify social status.

### Odor Stimuli and Delivery

Odor test stimuli were delivered to the exposed olfactory epithelium and consisted of the following (described in detail below): (1) L-Alanine (Ala; at concentration of 10^–5^ M made in RO-water; Sigma-Aldrich, St. Louis, MO, USA); (2) sulphated sex-steroid (5-androsten-3α-ol-17-one sulphate sodium salt; Steraloids, Inc., Newport, RI, USA, #A8470-000) at concentration of 10^−9^ M; (3) sulphated sex-steroid at concentration of 10^−12^ M; (4) female-conditioned water; (5) male-conditioned water; (6) control RO-water; 7) control 1% methanol.

The sulphated sex-steroid was chosen because previous EOG studies demonstrated that the *A*. *burtoni* olfactory system responds to conjugated (sulphated and glucurinated), but not unconjugated steroids^41^. While the identity of the compounds released by *A*. *burtoni* during social interactions are not yet known, males and females increase their urination in the presence of conspecifics and these types of conjugated steroids are likely released via urination rather than other routes (e.g. skin, gills)^33,34^. The abovementioned sulphated sex-steroid was chosen because the *A*. *burtoni* olfactory epithelium responds well to this compound and we were interested in how forebrain neurons responded to an example of a conjugated steroid as a comparison to the complex odor mixtures that were the focus of our interest. Whether or not this particular sex-steroid plays a role in social interactions in this species is not known. A sex-steroid stock solution of 10^−3^ M was made in 50% methanol:50% milliQ water, and then diluted to 10^−6^ M with 50% methanol:50% milliQ water followed by further dilution to the working solutions of 10^−9^ M and 10^−12^ M with 1% methanol:99% RO-water (test solutions prepared daily). Preliminary forebrain recordings showed responses to sex-steroid concentrations of 10^−9^ M and 10^−12^ M, and while both concentrations were tested for each fish, only 10^−9^ M was used for analysis because it provided more consistent results. The amino acid Ala was used as a putative non-social-related food odorant for comparison, but it is also possible that odors released by either sex could contain amino acids including Ala. Alanine also served as a reference control during recordings to verify the recording setup across individuals, and a concentration of 10^−5^ M was used based on our previous EOG recordings^42^. Stock solution of Ala (10 mM) was prepared weekly and test solution was prepared daily.

Two different socially-relevant complex odor samples were also tested: male-conditioned water and female-conditioned water. To generate these water samples, a 37.85 L tank was divided into three equally sized compartments with sealed clear acrylic barriers. On the morning of preparation (8–10 am), 4 L of RO-water and an air stone were placed into each compartment. A dominant male was placed in each of the left and middle compartments, and four gravid females were placed in the right compartment (chosen based on distended abdomens indicative of large oocytes). Fish were checked and observed for ~5 min every hour to verify that they were swimming normally and engaging in some level of social behaviors. This experimental paradigm mimics a natural mixed-sex population while allowing collection of separate water samples. Our previous work demonstrated that both males and females alter their urination rates in these social contexts^33,34^, and therefore, water samples likely contain compounds released via urine as well as other routes (e.g., skin, gills). All fish were then removed after 4 hours and water was collected from each compartment (5 samples per compartment of 30 ml each into 50 ml falcon tubes) and stored at −20 °C. Thus, female-conditioned water used as a stimulus was from four gravid females interacting with each other in the same compartment, as well as visually with dominant males across the barriers. Male-conditioned water used as a stimulus was from the dominant male interacting visually with another dominant male across the barrier on one side and a group of gravid females on the other side. The male-conditioned water samples were collected from dominant males different from those used for recording experiments. Each individual water sample was used for two experiments so that a dominant and subordinate male was exposed to the identical complex odor sample. Two different control solutions were also tested for each fish: RO-water and 1% methanol. RO-water was the control solvent for Ala, female water, and male water stimuli, and 1% methanol was the control solvent for the sulphated sex-steroid.

For the recordings, a constant flow (50 µl s^−1^) of control RO-water at 22 °C bathed the olfactory epithelium for several minutes, followed by 4 four-second applications of each of the 7 test stimuli presented in random order. This randomized delivery was verified here and in our previous EOG recording study not to influence olfactory responses when odors of different type or concentration were delivered sequentially^42^. Each individual fish received the same stimuli at the same concentrations, and RO-water (pH 8.1) served as the rinse solution between stimuli during all recordings. The stimulus duration of 4 s was chosen because our previous experiments testing different durations revealed that the responses showed minimal variance and plateaued at ~3–4 s, indicating that longer durations were not necessary, and that shorter durations were too variable. The stimuli were delivered by an 8-channel controlled gravity perfusion system with the same pressure across all individuals tested (VC3-8PG, ALA Scientific Instruments, Farmingdale, NY, USA). Stimulus solutions were delivered through separate tubes (MLF-8 millimanifold, ALA Scientific) and inter-stimulus intervals were 90–120 s. This inter-stimulus interval was used to match that used in our previous EOG recordings, and preliminary experiments here verified that EOG responses fully returned to baseline after odor application within this timeframe. Control RO-water was also tested intermittently throughout each recording period. A total of ~30–40 stimulus presentations (7 stimuli x 4 applications, with some repeats) occurred for each fish tested.

### Verification of Recording Sites

To verify that the recording sites were in the ventral telencephalon, electrolytic lesions were performed. Recordings were conducted as above with application of all test odors and control solutions, followed by current injection (5 µA, square 80 ms pulses at 10 Hz for 15 sec). Current injection to the gold and platinum-plated electrodes often also causes a small gold ball (~2–5 µm dia.) to deposit at the site, which can be histologically visualized. Brains were fixed in 4% paraformaldehyde (in 1XPBS), rinsed in 1XPBS, cryoprotected in 30% sucrose, sectioned coronally at 20 µm on a cryostat, and collected onto charged slides. Slides were dried overnight at room temperature and then stained with 0.1% cresyl violet, dehydrated in an alcohol series (50–100%), cleared in xylene, and coverslipped with cytoseal 60. Brain sections were examined for lesions with a Nikon Eclipse Ni microscope and photographs taken with a digital camera (Nikon DS-Fi2) controlled by Nikon NIS-Elements software (levels and contrast adjusted in Adobe Photoshop CS6).

### Data and statistical analysis

To distinguish whether or not each recorded neuron showed an excitatory response to a particular odor, we used the following response criterion: spike rate during stimulus application was 2 standard deviations above the spike rate measured during a 4 sec pre-stimulus time period. Thus, each neuron showed either a response or no response to each test stimulus. While we did encounter neurons in our multi-unit recordings that showed inhibitory responses to some odors, our recording protocol focused on isolating only excitatory responses and only these were analyzed. The percentage of neurons responding to each odor was compared between dominant and subordinate males with Fisher Exact tests. It is important to note that these forebrain recordings are difficult because many neurons are either silent or have slow resting discharge rates, making their discovery in the absence of odor application challenging. Although partial recording data were gathered from a greater number of neurons in additional fish (~25 more cells in 15 additional fish), only those neurons in which we recorded full responses (spikes and LFPs) from the entire suite of odors with clean and stable recordings (e.g. amplitude and frequency of spikes remained consistent in the control conditions with high signal to noise ratio, sufficient amplitude of the LFP) were used in the analysis and are reported here. Thus, approximately 50% of the recorded neurons met criteria for inclusion in statistical analyses. Responses with partial data recorded from these additional neurons are consistent, however, with the findings reported for those cells with complete data.

To examine the firing response properties of each neuron, the following characteristics of the spike response rate were analyzed for the 3 best responses (determined visually from the recordings to have high signal to noise ratio and spikes of consistent amplitude and shape) of each test compound (out of 4 tested for each neuron): pre-stimulus (4 sec period before stimulus application); stimulus (entire 4 sec period of stimulus application). Response duration was also measured as the time between the first spike after stimulus onset to the time of the last spike. For multiunit recordings, spike sorting was performed in spike 2 software prior to analysis. Spike rates during stimulus application were normalized to the pre-stimulus spike rates for each neuron [(stimulus spike rate-prestimulus spike rate)/prestimulus spike rate] and then compared between dominant and subordinate males using Generalized Linear Mixed Model (GLMM) tests.

We also used the LFP recordings to compare the power spectral densities of odor responses in dominant and subordinate males using Neuroexplorer software (NexTechnologies, Lexington, MA) as described previously^92^. For a subset of neurons (N = 7 per social status), we measured the maximum power spectral density % (averaged for 3 applications of each test odor for each neuron) at 3 different frequencies approximating theta (4–9 Hz), low beta (10–15 Hz), and high beta (16–28 Hz) oscillations during the second 2 seconds of the odor application, and compared them between dominant and subordinate males with two-way repeated measures (RM) ANOVA (neuron as repeated measure; status and odor type as factors). Differences in the spectral densities during stimulus application are indicative of odor-evoked neural activity in the vicinity of the recording electrode (~100–250 µm radius), and provide information on transfer functions related to integrative brain computations.

To analyze EOG recordings, we quantified peak amplitudes and slopes (slope of the initial negative phase was measured at 70% of peak amplitude in mV s^−1^) of the responses as in our previous study^42^. Each of these measures was calculated from three representative EOG waveforms from each test stimulus in each fish. Generalized linear mixed models (GLMMs) were used to compare EOG responses, with body size as a covariate.

Fish SL, BM, and GSI was compared between dominant and subordinate males with Student’s t-tests or Mann-Whitney rank sum tests. Dominant males (N = 7) had higher GSI values compared to subordinate males (N = 7) (Mann-Whitney Rank Sum test, U = 0.00, P < 0.001), but males of the different social states did not differ in size (SL: Student’s t-test, t = 0.237, P = 0.816; BM: Mann-Whitney Rank Sum test, U = 22.0, P = 0.805). All data were tested for normality and equal variance and those that did not meet these assumptions were either transformed or compared with non-parametric or mixed model tests. All statistical comparisons were made in SPSS 24 (IBM, Inc., Armonk, NY, USA) or SigmaPlot 12.3 (Systat Inc., San Jose, CA, USA).

## Data Availability

The datasets generated during and/or analyzed during the current study are available from the corresponding author on reasonable request.
